# Towards precision MRI biomarkers in epilepsy with normative modelling

**DOI:** 10.1093/brain/awaf090

**Published:** 2025-03-05

**Authors:** Remika Mito, James H Cole, Sila Genc, Graeme D Jackson, Andrew Zalesky

**Affiliations:** Department of Psychiatry, The University of Melbourne, Parkville, VIC 3052, Australia; Florey Department of Neuroscience and Mental Health, The University of Melbourne, Heidelberg, VIC 3084, Australia; Department of Computer Science, Hawkes Institute, University College London, London WC1V 6LJ, UK; Dementia Research Centre, Queen Square Institute of Neurology, University College London, London WC1N 3BG, UK; Department of Neurosurgery, Neuroscience Advanced Clinical Imaging Service (NACIS), Royal Children’s Hospital, Parkville, VIC 3052, Australia; Developmental Imaging, Murdoch Children’s Research Institute, Parkville, VIC 3052, Australia; Department of Paediatrics, University of Melbourne, Parkville, VIC 3052, Australia; Florey Department of Neuroscience and Mental Health, The University of Melbourne, Heidelberg, VIC 3084, Australia; Florey Institute of Neuroscience and Mental Health, Melbourne, VIC 3052, Australia; Department of Psychiatry, The University of Melbourne, Parkville, VIC 3052, Australia; Department of Biomedical Engineering, The University of Melbourne, Parkville, VIC 3052, Australia

**Keywords:** epilepsy, normative modelling, MRI biomarkers, focal cortical dysplasia, neuroimaging

## Abstract

Epilepsy is recognized as one of the leading targets for precision medicine, following on from the successes in cancer therapy, due to its substantial clinical heterogeneity and divergent therapeutic options. To bring personalized care to the epilepsies, there is a need for appropriate precision biomarkers that can identify disease processes or predict treatment outcomes at the individual patient level. Neuroimaging techniques, including MRI, have been transformative for clinical practice, particularly in medically refractory focal epilepsies. Advanced MRI techniques have the potential to bring precision medicine clearly into view for epileptology; however, there are challenges that must be overcome before cutting-edge neuroimaging tools can be used in clinical practice.

In this review article, we communicate our view that implementation of normative modelling frameworks will help to deliver robust quantitative MRI biomarkers for individualized prediction. Here, we provide recommendations for researchers and clinicians alike, from careful research design to clinical applications, that will help to identify diagnostic and predictive imaging biomarkers. Such precision markers will be key to delivering personalized medicine for the epilepsies.

## Teaser

Epilepsy is a key target for precision medicine due to its heterogeneity, but current research frameworks are often poorly suited to identifying biomarkers in heterogeneous cohorts. Mito *et al.* outline the potential of normative modelling, and argue that it could accelerate the delivery of much-needed neuroimaging biomarkers.

## Introduction

Epilepsy is one of the most common chronic neurological disorders, with an estimated 50 million individuals living with the condition worldwide.^1^ However, aggregating these millions of individuals under a single disease umbrella is potentially misleading; epilepsy encompasses a vast range of conditions, with the common feature being a predisposition to recurrent seizures. Perhaps in part due to this indiscriminate nomenclature, for decades the dominant pathway for therapeutic management of epilepsy has been a ‘one-size-fits-all’ approach, despite known heterogeneity in the underlying causes and mechanisms that drive seizures.^2^ Correspondingly, the proportion of individuals with medication-resistant epilepsy has remained virtually unchanged over the past 30 years (at ∼30%–40% of epilepsy patients), even with the explosion in novel molecular therapeutics.^3,4^ The need for individualized approaches to therapy has become increasingly evident, and epilepsy is now recognized as a leading target for precision medicine, following on from the successes in cancer therapy.^5^

Precision medicine is defined as treatment that is tailored to the needs of each individual patient, taking into account the specific causes of disease and targeting these for therapy.^6,7^ Although there has been an intellectual paradigm shift towards personalized approaches in the epilepsies, the focus of precision diagnostics and therapy has been largely limited to targeting specific genetic causes,^2,5,7-11^ despite monogenic epilepsies representing only a small subset of all epilepsy patients.^12^ Precision care need not be limited to those with genetically defined epilepsies. To deliver personalized care for all, there is a need for appropriate precision diagnostics that extend beyond genetics and include the neuroimaging phenotype, electroclinical signature, demographic characteristics, neuropsychological profile and comorbidities of a given individual. Amongst these, MRI stands out as an important precision tool for diagnosis, particularly in individuals with medically refractory epilepsy for whom disease burden and associated risks can be particularly substantial.^13-15^ Although the future of epilepsy precision care will undoubtedly require multi-dimensional approaches that incorporate multiple phenotypic characteristics of an individual, we focus here on the major strides needed in neuroimaging research to deliver personalized tools for clinical use in epilepsy.

In this review article, we discuss the potential of MRI biomarkers as precision tools for epilepsy, particularly in the context of medically refractory focal epilepsies. We highlight the current state-of-the-art MRI tools that demonstrate promise in epilepsy research studies, and the major challenges in translating these research tools into clinical use and practice. Here, we focus on MRI techniques that could be transformed into powerful diagnostic or prognostic biomarkers. We provide recommendations for epilepsy neuroimaging research moving forward, with a particular focus on normative modelling frameworks that may accelerate the development of valuable MRI biomarkers for precision medicine in epilepsy.

## Epilepsy: a heterogeneous clinical condition

**Figure 1:**
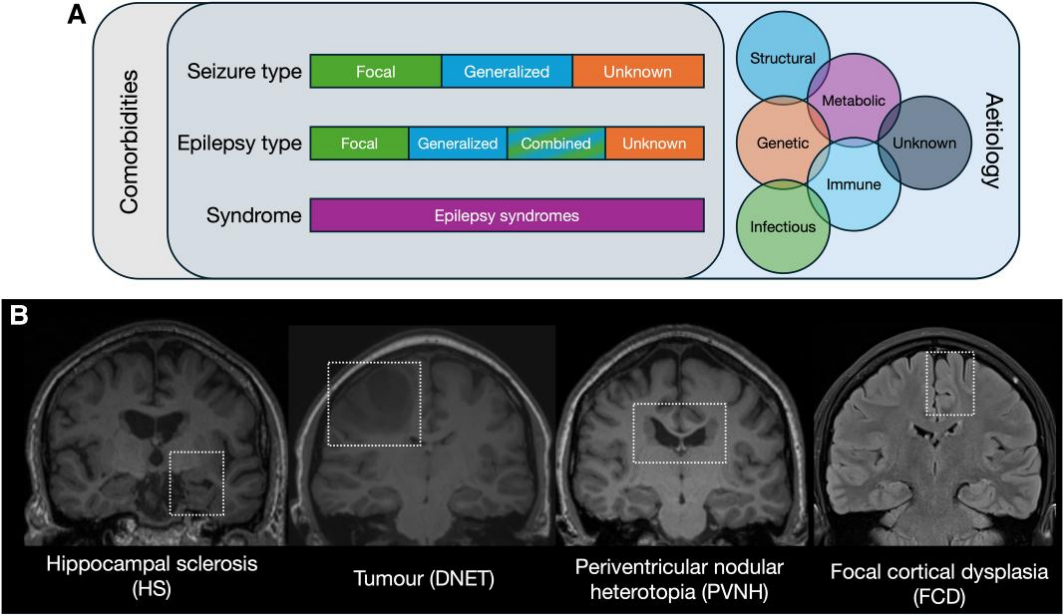
**Challenge of heterogeneity in epilepsy**. (**A**) Classification schema for epilepsy. The heterogeneity of epilepsy is well captured by the complex classification schema that are used when an individual is diagnosed with epilepsy. Epilepsies are classified at three levels (seizure type, epilepsy type and epilepsy syndrome). In addition, the aetiological causes of epilepsy are wide ranging, and comorbidities should also be considered given their impact on prognosis and treatment. (**B**) Common structural causes of epilepsy. There are numerous potential structural causes to seizures including, from *left* to *right*: hippocampal sclerosis (HS), tumour [dysembryoplastic neuroepithelial tumour (DNET)], periventricular nodular heterotopia (PVNH), and focal cortical dysplasia (FCD). For these individuals, neuroimaging is essential, as it can help to identify the cause of epilepsy seizures.

The sheer heterogeneity of epilepsy is aptly captured by the complex classification schema that are used when an individual is diagnosed with the condition. Epilepsies are classified at three levels: by seizure type, by epilepsy type and by syndrome (Fig. 1). At each of these levels, there are multiple types and syndromes defined (e.g. focal, generalized or unknown seizure types and defined syndromes such as Lennox-Gastaut syndrome or juvenile myoclonus epilepsy). In addition, seizures can result from almost any perturbation to brain function, resulting in a wide spectrum of causes of epilepsy.^16^ There are six defined categories of potential causes: structural, genetic, metabolic, infectious, immune, and unknown; and these varying causes likely have some level of overlap in a given individual.^17,18^ Within each of these categories, there is still substantial variability in the underlying pathogenesis; e.g. structural causes range from prenatal injuries or malformations during development, to tumours, strokes and brain injury in adulthood (Fig. 1B). To add to the complexity, epilepsy patients may have various comorbidities, including learning difficulties or neuropsychiatric disorders (such as autism spectrum disorder or depression), and these comorbidities may affect prognosis as well as treatment efficacy.^17,19^ All these elements together (seizure type, epilepsy type, epilepsy syndrome, causes and comorbidities) constitute the framework for International League Against Epilepsy (ILAE) epilepsy classification.^18,20^

When an individual first presents to a seizure clinic, a range of clinical examinations may be performed to identify the cause of seizures and/or make a diagnosis of epilepsy. After first attempting to distinguish potential epilepsy seizures from seizure mimics, a full diagnostic work-up should be performed, which will generally include evaluation of an individual’s medical history, physical and neurological examination, EEG, neuroimaging (including MRI), and laboratory tests, including genetic testing.^17^ Through this detailed assessment process, the goal is to identify the specific epilepsy aetiology, in order to guide treatment planning for a given individual. Given the substantial variability in epilepsy conditions, this process can be lengthy and clouded with uncertainty, particularly in clinical settings where the vast range of recommended tools may not be readily available.

The first line of treatment for epilepsy patients is anti-seizure medications, and the choice of medication should be guided by an individual’s specific epilepsy diagnosis, and by their demographic characteristics or comorbidities.^21^ Although there are now over 25 approved medications [US Food and Drug Administration (FDA)-approved], approximately a third of epilepsy patients are considered medically refractory, meaning that they cannot achieve seizure control with available pharmacological treatment. Neuroimaging can be particularly beneficial for patients with medically-resistant seizures, as these are commonly due to structural abnormalities. Surgical removal of pathological brain tissue offers the best chance of seizure control for these individuals.^22-24^ However, the success of epilepsy surgery depends on the ability to localize a lesion on MRI.^25-28^

We therefore focus in this review on the potential for MRI biomarkers in the diagnosis of structural causes of epilepsy, where improved identification of lesions could be transformative. However, the use of MRI in epilepsy extends beyond identification of lesions into a potential prognostic tool to track treatment efficacy or predict response across all epilepsies, particularly through multi-modal imaging frameworks. The next section highlights the potential of MRI as both a diagnostic and prognostic tool for precision epileptology.

## The potential of MRI as a precision tool for epilepsy

**Figure 2:**
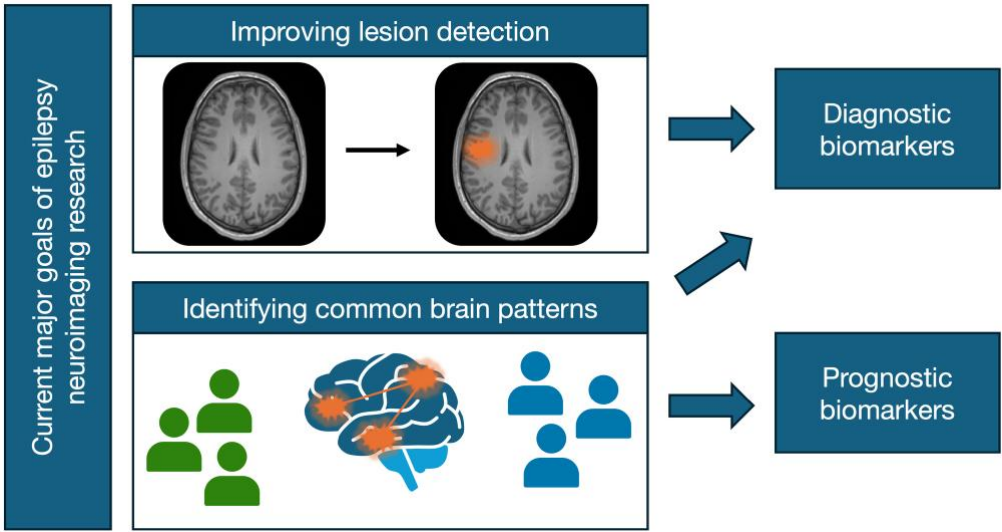
**Current approaches for neuroimaging research in epilepsy**. Epilepsy MRI research studies have been focused on addressing one of two major goals: (i) improving identification of epileptogenic lesions in individual patients; or (ii) identifying common brain patterns in groups of patients. Novel diagnostic biomarkers could be developed by translating research tools that focus on improving identification of structural causes of epilepsy, which currently rely largely on identification via visual inspection. Brain imaging patterns that characterize specific subgroups of epilepsy could also be used as diagnostic tools. However, such imaging tools are likely to make more valuable prognostic biomarkers if they are able to differentiate different response or progression groups of patients.

Neuroimaging techniques have advanced rapidly since the advent of MRI scanners in the 1980s, providing a window into the *in vivo* structure and function of the human brain. Broadly speaking, epilepsy MRI research over the past two decades has been focused on addressing one of two major goals: either to improve identification of epileptogenic brain tissue in individual patients, or to identify common neural patterns in groups of patients. In theory, both approaches could lead to precision biomarkers: either in identifying the underlying structural cause of epilepsy (i.e. diagnostic biomarkers), or in identifying a brain pattern that is characteristic to a particular response group of patients or particular clinical progression or severity (i.e. prognostic biomarkers) (Fig. 2). In both cases, research studies have progressively advanced through implementation of novel developments in MRI acquisition, postprocessing and analysis tools, as they have become available. Here, we briefly review the major advances in MRI research for both lesion detection and epilepsy-specific brain patterns, and how these advancements may be valuable in precision medicine in future. We focus on MRI modalities or techniques that are particularly suitable for translation into diagnostic or prognostic biomarkers through normative modelling frameworks. Although there are other common imaging approaches that are used in epileptology for specific purposes such as surgical planning (Box 1), these are beyond the scope of this review.

Box 1 Key imaging techniques used in epileptology

**T_1_-weighted imaging:** Structural image that provides tissue contrast based on T_1_ relaxation time. Used for visual inspection as it emphasizes anatomical structures and can be used to derive quantitative measures (e.g. volumetry and surface-based measures).

**Voxel-based morphometry:** Statistical approach for comparing quantitative differences between groups in measures derived from structural MRI. Can be used to identify differences in brain shape or size common to a group of patients.

**Surface-based morphometry:** Similar to voxel-based morphometry, but uses imaging features that relate to the brain’s surface (e.g. cortical thickness). Can be used to identify more subtle changes in brain anatomy, e.g. in focal cortical dysplasia.

**Volumetry:** An approach to measure the volume of a specific structure or region of interest from structural MRI. Useful for identifying hippocampal volume to assess asymmetry or atrophy.

**T_2_-weighted imaging:** Structural image that provides contrast based on T_2_ relaxation time. Typically used to visualize pathological abnormalities including epileptogenic lesions (e.g. hippocampal sclerosis, focal cortical dysplasia).

**Fluid-attenuated inversion recovery (FLAIR):** T_2_-weighted structural image that has CSF signal suppressed. Used for improved visualization of pathological abnormalities (e.g. hippocampal sclerosis, focal cortical dysplasia).

**T_2_ relaxometry:** A quantitative technique that produces maps of T_2_ relaxation. Demonstrates improved sensitivity to identify hippocampal sclerosis.

**Diffusion-weighted imaging (DWI):** MRI that is sensitive to the diffusion properties of tissue. By acquiring directionally encoded diffusion-weighted images, measures relating to the brain’s white matter microstructure can be derived. These measures may help to better identify subtle epileptogenic lesions. DWI can also be used for tractography in surgical planning (see below).

**Tractography:** DWI can be used to map the brain’s white matter tracts. Tractography can be used in surgical planning, to avoid cutting white matter pathways associated with key functions (e.g. avoiding Meyer’s loop to minimize visual deficit during anterior temporal lobectomy).

**Functional MRI (fMRI):** fMRI is sensitive to changes associated with blood flow, and can be used to infer time-dependent changes in brain activity. It can be used to identify brain networks associated with specific functions (task-fMRI; see below), or to quantify measures relating the brain’s resting state functional connectivity (rs-fMRI). Functional connectivity measures may help to predict surgical outcome in epilepsy patients.

**Language or motor fMRI:** Task-based fMRI using language or motor tasks can be used to identify brain networks associated with language or motor function. Language fMRI is commonly used to identify language lateralization in epilepsy patients, or guide surgical planning for resections close to eloquent cortex. Similarly (though less commonly), motor function can be mapped to avoid deficits following resections close to motor cortex.

**Combined EEG and fMRI:** Simultaneous EEG and fMRI that enables mapping of haemodynamic networks related to electrical brain activity. Typically used to assess brain networks related to epileptiform discharges, providing localization of epileptogenic foci.

**Susceptibility-weighted imaging (SWI):** MRI technique that is sensitive to paramagnetic and diamagnetic properties of tissue. Can improve the identification of cavernomas.

### MRI as a diagnostic biomarker for lesional epilepsies

Precision medicine requires precision diagnostics, and there is little doubt that MRI holds enormous potential as a key diagnostic tool in epilepsy. Indeed, MRI has already revolutionized clinical practice in epilepsy: it has enabled the identification of structural lesions that drive seizure activity in focal epilepsy and is routinely used to delineate lesion boundaries and their relationship to eloquent cortex in surgical planning.^29^ The use of MRI in epilepsy is a success story for its clinical translatability in neurological conditions. Not only has neuroimaging helped to identify clear structural causes of epilepsy (such as acute ischaemic stroke and brain tumours), but identification of epileptogenic lesions has improved with incremental advancements in MRI research. The difficulty of detecting subtle lesions, coupled with the potentially transformative clinical significance of lesion identification, has meant that epileptology has often been at the forefront of adopting the latest developments in neuroimaging into clinical practice.

Over time, we have seen the application of contemporary MRI techniques help to identify increasingly subtle epileptogenic lesions, right from the revolutionary introduction of the imaging technique.^30-32^ Early advancements came from improved visual detection, thanks to optimized image contrasts, and increasing image quality and resolution. For example, it was not until the 1990s that hippocampal sclerosis was able to be reliably detected on MRI,^33,34^ enabled thanks to advances in MRI hardware, sequences and optimal scanning planes. Histological validation of these MRI-detected abnormalities has supported the use of imaging tools as a robust diagnostic biomarker for hippocampal sclerosis and pathology.^35-38^ At the time the Palmini classifications were published, mild malformations of cortical development were still thought unable to be detected by MRI.^39^ However, even the subtle features of focal cortical dysplasias (FCD; with imaging features including T_2_/FLAIR hyperintensity and grey-white matter blurring^40-42^) have since been demonstrated to be identifiable on MRI with histopathological validation,^43,44^ thanks to improved spatial resolution and signal-to-noise ratio (SNR). Indeed, identification of epileptogenic lesions on MRI depends on a number of factors, including the expertise of neuroradiologists who assess the scans,^45^ imaging hardware characteristics like field strength^46-48^ and the sequences acquired.^49-51^ MRI is now recommended in all individuals with new-onset seizures (except those with idiopathic generalized epilepsies), and current recommendations propose the use of standardized high-resolution structural sequences across all epilepsy centres to maximize the likelihood of detecting subtle epileptogenic lesions.^29^

Despite huge improvements in the ability to detect epileptogenic lesions, there remain limitations to relying on visual inspection alone. As such, quantitative imaging and postprocessing approaches have been widely adopted in epilepsy research studies, to potentially improve lesion detection. For hippocampal sclerosis, although visual inspection on high-quality MRI is generally considered sufficient,^52,53^ quantitative approaches like T_2_ relaxometry or mapping^54,55^ or hippocampal volumetry,^56-58^ have long demonstrated improved or additional sensitivity to visual inspection, as have quantitative analysis approaches on image intensity.^59,60^ Recent T_1_ mapping approaches with magnetization prepared 2 rapid acquisition gradient echo (MP2RAGE)^61^ also demonstrate sensitivity to hippocampal sclerosis,^62^ due to their enhanced ability to delineate tissue boundaries, with potential to detect layer-specific changes further enhanced at ultra-high field.^63^ The use of quantitative apparent diffusion coefficient (ADC) indices also demonstrate ability to lateralize and localize hippocampal sclerosis, potentially more so than hardware changes like increasing field strength.^51^

In the case of more subtle epileptogenic lesions like FCDs, where visually appreciable imaging signs exhibit variable sensitivity and specificity,^64^ quantitative approaches have been particularly important. For structural MRI, the use of surface-based morphometry has demonstrated increased sensitivity to detecting FCDs,^65-68^ more so than voxel-based morphometry^69^ or intensity-based analysis methods.^55^ The use of MP2RAGE sequences can also improve sensitivity and specificity of FCD detection compared to MPRAGE.^70^ In addition to relying on structural or morphological features, probing tissue microstructure using diffusion MRI adds potential value in detecting or localizing subtle lesions^71-74^ at the individual patient level, with advanced or compartment-based diffusion models like fixel-based analysis (FBA^75^) or neurite orientation dispersion and density imaging (NODDI^76,77^) demonstrating promise for FCD localization. Mapping brain function using functional MRI (fMRI), either independently using functional connectivity measures^78,79^ or in combination with EEG (EEG-fMRI^80^) shows variable promise with localizing epileptogenic lesions. The development of magnetic resonance fingerprinting (MRF) protocols to simultaneously provide quantitative tissue property maps (including T_1_, T_2_, proton density and tissue fraction maps) also demonstrates potential for improved detection of subtle lesions with clinically feasible scan times.^81^ Combining MRI-based quantitative measures into multimodal approaches also demonstrates promise.^82^

As will be discussed later, one of the key challenges with implementing quantitative neuroimaging approaches into clinical practice is that these approaches inherently require some interpretation of their absolute values, either by comparison to the contralateral (presumably unaffected) hemisphere in a given individual, or to a healthy reference cohort. For this reason, although some research-affiliated hospitals have been able to make use of quantitative tools, the use of these MRI techniques is still very limited in widespread clinical practice.

### Identifying common brain patterns in epilepsy: potential as predictive biomarker?

Alongside developments in lesion detection, there has been a wealth of neuroimaging literature examining brain abnormalities in epilepsy conditions at the group level. These research studies typically report statistically significant differences in a given MRI measurement in epilepsy cohorts, when compared to a control cohort. For example, even in focal epilepsies, studies have reported widespread functional and structural abnormality across the brain, in measures like cortical thickness quantified from structural MRI,^83^ microstructural changes in white matter quantified using diffusion-weighted imaging (DWI)^84^ and functional connectivity quantified by resting state fMRI.^85^ Together, these neuroimaging techniques have helped to grow our understanding of common brain changes in the epilepsies, driving the conceptualization of epilepsy as a brain network disorder.^86,87^ However, it is important to note that statistical significance does not equate clinical importance, and these findings on their own are largely uninformative for assessing an individual patient’s potential clinical outcome. Indeed, these neural abnormalities will only translate into clinically useful biomarkers if they have predictive value; that is, they are accurate proxies for an individual’s diagnosis, prognosis or treatment response.^88^

To this end, research studies have explored the predictive value of MRI-derived measures in epilepsy. A common approach is to assess the accuracy of predicting postsurgical seizure outcome from presurgical imaging features.^89-94^ MRI features have also been used to predict medication or treatment response, often alongside clinical information,^95-97^ or to predict existing clinical phenotypes like language impairment.^98^ In such studies, the goal is to identify certain brain patterns that are characteristic to a given clinical subgroup or outcome group. Increasingly, machine learning approaches have been adopted in these studies, often implementing classification or clustering based approaches to distinguish different groups.

## Current challenges in developing MRI precision markers

Despite the promise of MRI as both a diagnostic and predictive tool in epilepsy, there has long been a translational gap from research method to clinical practice. Discussing all the potential barriers to clinical translation is beyond the scope of this review; however, there are two major challenges that could be specifically addressed through targeted research design and analysis. As such, in this section, we highlight these two major challenges that have hampered development of MRI-based biomarkers in epilepsy: (i) the challenge of disease heterogeneity; and (ii) challenges associated with developing novel (quantitative) MRI biomarkers.

### The challenge of heterogeneity in epilepsy

Given the palpable complexity in the classification schema for the epilepsies (Section 1 in Fig. 1), it is no surprise that we face difficulty when searching for novel MRI biomarkers—clinical heterogeneity poses challenges for research design. The first challenge is in defining the clinical group of interest: commonly, subgroups are based on a categorized phenotype (e.g. focal or idiopathic generalized epilepsy), or on a given structural cause. However, even within these defined subtypes, there can be substantial variability. For instance, in taking a specific structural cause of focal epilepsy, such as focal cortical dysplasia, there is still spatial variability in the location of epileptogenic zone, as well as differences in histopathological classification, all of which may affect clinical phenotype as well as treatment outcome. Similarly, temporal lobe epilepsies caused by hippocampal sclerosis demonstrate differential severity of structural brain changes depending on the laterality of epilepsy (left versus right).^83,99^ Heterogeneity within epilepsy phenotypes has undoubtedly limited our understanding of the pathophysiological changes that underpin the epilepsies.^100^

Another reason that heterogeneity in clinical cohorts has long posed a major issue for research studies is that classical statistical frameworks rely on comparisons of group means. These conventional case-control comparisons assume that there is a common pathophysiological process that similarly affects all individuals within a clinical group. In the context of MRI studies, when deriving an imaging signature of epilepsy (or of a particular subtype), there is an assumption that a single imaging pattern can differentiate epilepsy cases from healthy control participants.^101^

The patterns common to a clinical group when compared to a control group may reflect the underlying neurobiological processes at play. However, when the clinical group of interest is heterogeneous, the group-level patterns may not be representative of any given individual’s neuroimaging profile. For example, large multicentre studies, such as ENIGMA, have demonstrated common grey and white matter changes in ‘all’ epilepsies,^83,84,102^ and suggest that there are common neuroanatomical signatures of epilepsy that are shared across syndromes, in support of network-based models of epilepsy.^103,104^ Although conceptualizing common patterns across the epilepsies is valuable, there has been little success in better understanding the pathophysiological mechanisms as a result of these approaches.

If we rely on group-level findings for MRI-based biomarker development, we may fail to take into account individual-level differences, which are more likely to be meaningfully associated with disease processes and treatment response. Indeed, capturing disease hallmarks in the presence of heterogeneity will not only help to understand disease effects, but could also provide more personalized approaches to treatment through more accurate diagnosis and prognostication.^101^ As will be discussed in the following section, MRI holds enormous promise as both a diagnostic and predictive tool in epilepsy; however, there remain translational gaps from research method to clinical practice.

### The challenges associated with translation of novel MRI biomarkers

Despite clinical heterogeneity, the development of MRI-based biomarkers is both practical and potentially rapidly implementable in the context of epilepsy, where neuroimaging is a routine part of the clinical work-up in newly diagnosed patients. Currently, the use of MRI in clinical epileptology is largely qualitative, relying on visual evaluation of magnetic resonance images by a radiologist, while the most common quantitative approaches are geared towards characterizing the morphology of the hippocampus (e.g. with volumetry and T_2_ relaxometry used clinically to diagnose hippocampal sclerosis). Although radiological reading of MRI is generally sufficient in many clinical contexts, in the case of epilepsy, visual evaluation of MRI scans can be limited due to the subtle nature of brain abnormalities.^64^ To improve diagnosis and prognosis in epilepsy, the use of advanced and quantitative MRI approaches shows tremendous promise.

Since the development of MRI, quantitative techniques have been the focus for most MRI scientists and physicists: in theory, MRI could be used to measure almost any physiological property,^105^ meaning that it holds enormous potential for probing brain structure and function (as well as other tissues) *in vivo*. For clinical research, the interest in quantitative approaches has evolved alongside an increased focus on precision medicine, with growing understanding of their potential to improve diagnosis and prognosis in disease.^106^ Despite mounting interest and substantial research into quantitative MRI (qMRI) techniques and their application to disease over the past two decades, the use of qMRI measures in clinical decision-making and routine practice is still very limited.^107,108^

There are a number of key barriers to the clinical translation of quantitative MRI measurements into precision biomarkers. Quantitative MRI contrasts generally require at least two (and usually many) volumes to be acquired to map tissue properties, along with modelling of the data to extract meaningful measures (e.g. varying echo time in T_2_ relaxometry, or varying diffusion weightings and directions in DWI). This means that qMRI measurements are generally more lengthy and costly to acquire and analyse than conventional clinical images. Despite the level of postprocessing required, deriving quantitative measures from clinical MRI data (e.g. volumetry or morphometry) is, however, thought to offer favourable cost-benefit ratio in the context of lesional epilepsies.^64^ For quantitative imaging approaches, such as DWI, improvements in scanner hardware and multi-band and accelerated sequences have made acquisition much more clinically feasible; however, there remain other challenges that prevent widespread clinical use.

A key problem for the use of qMRI approaches is that the derived measurements are highly dependent on scanner hardware and software, and on acquisition protocols. For visual inspection of conventional standard-of-care images, these differences are generally tolerable, as long as radiologists are able to appreciate abnormalities from a given image.^106^ However, for quantitative imaging, variations in MRI vendors and field strength, scanner models and software versions, scanner age, radiofrequency (RF) coils, along with the countless adjustable acquisition parameters, all contribute potential sources of variability to the measurement of interest. Such technical or methodological variability can overshadow meaningful biological variability, while also making quantitative MRI suffer from substantial reproducibility issues.^105^ This makes it not only incredibly difficult to compare qMRI measurements across different clinical settings or sites, but additionally very difficult to establish any threshold or cut-off values for abnormality that would enable translation into biomarkers.

Indeed, it is important to highlight that the measurements that might be derived from quantitative imaging cannot be understood in isolation: they require context, either through the use of benchmark or threshold cut-offs, or through comparison against normative data. For example, to assess structural abnormalities that might be indicative of focal cortical dysplasia, researchers have adapted voxel-based morphometry pipelines to compare structural MRI scans from individual patients relative to control cohorts.^69,109-111^ Here, the use of (often arbitrary) significance threshold values that are computed relative to the control cohort have demonstrated varying levels of success. The use of quantitative analysis approaches that require a tissue segmentation step are potentially confounded by the presence of signal changes that characterize lesional tissue,^69^ posing a challenge for the use of such methods as robust biomarkers. The use of machine learning approaches, combined with statistical approaches that can model site differences,^112^ demonstrates promise in the development of diagnostic imaging markers from surface-based measures, for example, in the detection of focal cortical dysplasia.^68^ However, such approaches require control data from a given site in order to model these site effects, limiting their generalizability. To develop robust and generalizable imaging biomarkers, pooling data from multiple sites and studies is becoming increasingly common, particularly for machine-learning based applications. However, site-related effects, not only in MRI-based measures, but additionally those inherent to different clinical settings (e.g. clinical referral patterns, treatment patterns, measured outcomes, etc.) pose a significant challenge. Standardized neuroimaging protocols and processing standards would be hugely valuable in this context, while adherence to consensus recommendation for clinical reporting^113^ could ensure relevant outcome variables are available across multiple sites for robust prediction. Data harmonization approaches will be key when using multi-site qMRI data; however, the potential risks of data leakage when using batch harmonization approaches on neuroimaging-derived features should not be underestimated.^114,115^

Another potential issue is that many of these quantitative analysis approaches have been developed for group comparison, and may in fact be ill-posed for identifying individual abnormalities.^116^ Although techniques like voxel-based or surface-based morphometry, as well as more complex diffusion-based analyses,^75^ have been adapted for identification of individual-level abnormalities in epilepsy, the need for substantial postprocessing, including derivation of measures through a template normalization step makes it inherently difficult to translate beyond use at a single site. Clinical translation of quantitative MRI is likely better suited to approaches that can be directly integrated into scanner systems, or automated via commercial platforms, as indeed commercial routes have demonstrated the most success for clinical translation of MRI biomarkers.^106^

## Normative modelling approaches: a potential solution

Developing precision imaging biomarkers in the context of clinical heterogeneity necessitates the use of large datasets and modelling approaches that can overcome the aforementioned challenges. To this end, we are at an opportune time, where both huge amounts of openly accessible neuroimaging datasets are available (thanks to the open access ethos of the neuroimaging community), as are statistical and machine learning approaches that can make sense of complex and quantitative measures. Large consortia studies, like ENIGMA-Epilepsy, will undoubtedly be hugely valuable in the quest for generalizable imaging biomarkers, as will the application of algorithms that can parse clinical heterogeneity to make meaningful predictions at the individual level. Artificial intelligence approaches have generated growing interest for their potential to revolutionize diagnosis and treatment in epilepsy, although translation into clinical practice lags behind.^117-119^

**Figure 3:**
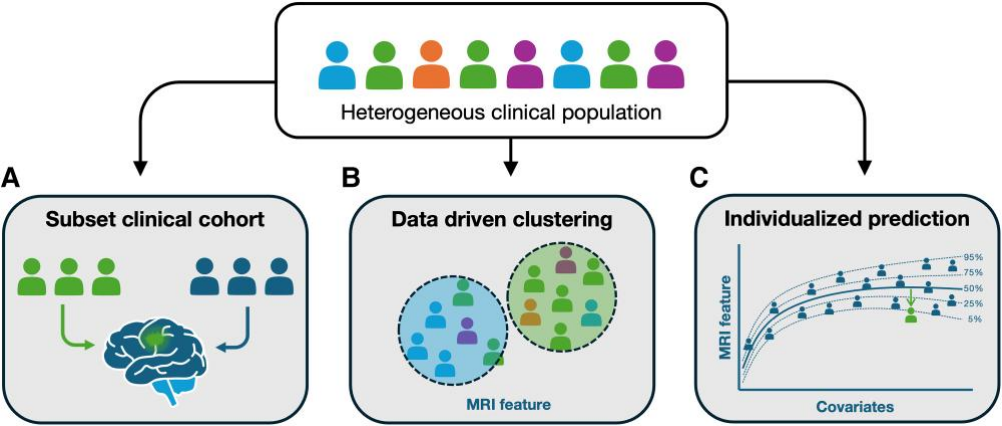
**Common and emerging frameworks for identifying imaging biomarkers in heterogeneous clinical cohorts**. (**A**) Heterogeneous clinical populations may be categorized based on a given clinical category, to determine if there are characteristics specific to a given clinical subtype (e.g. in epilepsy, comparing patients with focal versus generalized seizure types). (**B**) Alternatively, imaging features can be used to cluster participants into subtypes, and these different subtypes may be associated with distinct clinical outcomes. However, there may still be clinical heterogeneity within a given cluster. (**C**) One of the limitations to subtyping or clustering approaches is that they assume homogeneity within a given subtype or cluster. Therefore, individualized approaches may be more valuable in heterogeneous clinical populations, particularly for the development of precision biomarkers, which should take into account the specific characteristics of an individual.

To address the challenge of clinical heterogeneity, there are a number of common and emerging frameworks that have been implemented in epilepsy MRI research studies (Fig. 3). These can be broadly differentiated into approaches that make use of existing clinical labels to separate or subset heterogeneous cohorts (i.e. group comparisons), and data-driven approaches that rely on imaging phenotypes to distinguish meaningful clusters of patients. Early work focused on using clinical labels to differentiate subgroups in epilepsy, identifying brain patterns that may be characteristic to different groups. However, with the move toward designing neuroimaging-based diagnostic or prognostic tools, the goal has shifted from identifying common patterns, to single-subject prediction. Here, the most common approach has been to adopt data-driven clustering methods that may enable individual subjects to be classified into one or more groups (see Sone and Beheshti^118^ for a review).

Although such approaches are promising, grouping or clustering techniques by design still assume homogeneity within clusters, which may not always be a valid assumption.^120^ This limitation has motivated researchers to adopt approaches that can characterize individuals based on latent disease factors^121^; i.e. rather than belonging to a specific subgroup, a given patient may show varying levels of different disease factors. Models trained on such factors can then be used to predict clinical outcomes (e.g. drug response or postsurgical seizure outcome) by taking into account interindividual variability.

### Normative models for MRI in epilepsy

A potentially more interpretable approach for individual prediction is normative modelling. Normative modelling involves mapping the normal distribution of a measure or feature of interest according to a clinically relevant characteristic (e.g. age or sex) in a given population, then subsequently assessing how much an individual deviates from this normative range (Box 2). For neuroimaging-derived features, normative models can be used to estimate variation within defined brain regions, or at each individual imaging voxel within a common space. Such approaches have gained substantial interest in psychiatry and dementia, where clinical heterogeneity is similarly a key characteristic.^120,122-128^

Box 2 What is normative modelling?

Normative models are statistical modelling techniques that can map the trajectory of a given measure against clinical variables of interest as well as age, sex and other demographic features. By charting normal variability in a given measure, individual patients can then be benchmarked against the normative reference defined by healthy comparison individuals of the same age, sex, etc. Deviations from the expected range can thus be quantified and spatially localized. Such approaches offer a robust way to study patient differences as they can provide statistical inferences at the level of the individual. Of note, normative modelling approaches focus on modelling variation at a population level, meaning that measures of uncertainty can also be provided, which could be useful for clinical decision-making.

**Figure 4:**
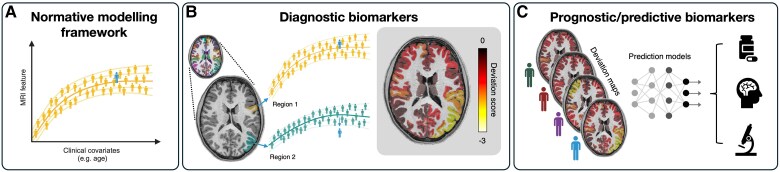
**Normative modelling for diagnostic and prognostic biomarkers in epilepsy**. (**A**) The normative modelling framework can be used to map the trajectory of MRI features [e.g. cortical thickness (from structural MRI) or fractional anisotropy (FA) (from diffusion weighted imaging, DWI) across voxels or regions] across a clinical variable (most commonly, age). Individual patients can then be benchmarked against this normative reference. (**B**) Normative modelling approaches could be used for diagnostic biomarker development in focal abnormality detection. For example, normative models are often generated for distinct brain regions, and where a given patient has a focal abnormality, they may exhibit deviation from the norm in one region, but not in others. By mapping deviation scores for a given individual across all brain regions (or brain voxels), we can obtain deviation maps, which may help to localize focal lesions. Such maps could be created using any quantitative MRI feature, making it particularly useful for translating quantitative MRI tools into biomarkers. (**C**) In turn, deviation maps themselves could be used as inputs into prediction models (e.g. for identifying efficacious treatments, cognitive or clinical outcomes, or pathological subtypes). Part of this figure was created in BioRender. Mito, R. (2025). https://BioRender.com/l77q099.

A common example of such normative models is in paediatric growth charts, which can be used to track a child’s development by benchmarking their height or weight against their age. In a similar manner, normative models can be applied to neuroimaging-derived features, for example, to map the trajectory of brain volume or cortical thickness against clinical variables of interest (such as age and sex; see Fig. 4A). When benchmarking a patient against the normative model, outputs include a predicted brain score, predicted variance, and a deviation score or *Z*-score. Such models can also be used across brain regions, with each brain region modelled separately to provide *Z*-score maps for a given individual. Such *Z*-score maps could be valuable in the context of lesion detection in epilepsy, whereby an individual patient might exhibit deviation from the norm in a given brain region (around the epilepsy lesion), but not in other brain regions (Fig. 4B). In addition, these *Z*-scores could be used in prediction models that aim to assess an individual patient’s treatment outcome or prognosis (Fig. 4C). By using such deviation scores to predict outcome measures, we could then extract predictive value from these *Z*-scores. This makes for a much more interpretable prediction measure than other promising scores, which are commonly based on disease factors or components.

There are a number of different tools available for modelling normative data. At the simplest level, linear models may be appropriate for mapping the trajectory of a given brain feature along a clinical covariate of interest. However, more commonly, non-linear approaches are used. Common approaches have been summarized in key papers^122^ and include models like Gaussian Process regression, Bayesian Linear Regression, Hierarchical Bayesian Regression, and Generalized additive models. Normative models have now been applied to develop large-scale brain charts across the lifespan,^125^ enabling benchmarking of individual brain MRI-derived features against population-level norms. Established tools, such as the BrainChart app, enable interactive computation of deviation scores for new patient scans using the largest normative database of MRI scans compiled to data (https://brainchart.shinyapps.io/brainchart/).

Normative models often cross-sectionally map age-related brain features but can also be mapped longitudinally to provide improved age-related trajectories. Longitudinal modelling can enable tracking of disease progression over time, as well as monitoring of potential normalization of aberrant brain deviations in response to drug therapy or other interventions. Given that cross-sectional normative models are prone to underestimating rates of change in deviation scores,^129^ longitudinal modelling will be key to accurately charting individual deviations across time. Longitudinal models are usually trained using data from the first acquisition time point (due to sample size considerations) and the trained model is then applied to measure individual deviations at subsequent follow-up assessments.^130^ Such longitudinal approaches have been used to map progression of imaging features over time in clinical cohorts (e.g. demonstrating attenuation of cortical thickness abnormalities in schizophrenia over a 10-year period^131^), as well as to map cognitive decline (e.g. in preclinical Alzheimer’s disease based on cognition at follow-up deviating from expectations established at baseline^132^).

For epilepsy, normative modelling approaches could similarly be used to accelerate development of precision biomarkers. The concept of normative models—i.e. to compare an individual against a healthy or ‘normal’ reference—has widely been used in epilepsy neuroimaging studies to identify individual patient abnormalities, as well as to map anomalies from EEG data.^133,134^ However, these normative frameworks have been furthered in recent years to estimate population-level distribution of imaging features, conditioned on age or other clinical/demographic characteristics of interest, using more complex statistical and/or machine learning frameworks. The implementation of such frameworks could enable development of novel diagnostic tools for anomaly detection in epilepsy, as well as prognostic tools for clinical prediction.

#### Normative modelling for anomaly detection or localization

A common example for normative benchmarking in epilepsy is in assessing hippocampal volumes for the identification of hippocampal sclerosis or hippocampal abnormalities in temporal lobe epilepsy. By having a reference dataset, hippocampal volumes of individual patients can be converted into *z*-scores to assess any deviation with respect to the norm. To define hippocampal abnormality or atrophy, thresholds are commonly used [e.g. below the first percentile^135^ or two standard deviations (SD) from the control mean^136-139^]. To translate hippocampal volumetry into a valuable clinical tool, it must then demonstrate the ability to appropriately detect hippocampal atrophy in patients (i.e. correctly classify patients with hippocampal atrophy with high sensitivity) while minimizing false positives (i.e. incorrectly classifying those without hippocampal atrophy). Importantly, for large normative reference sets, the use of automated approaches to assess clinical measures (i.e. in this case, automated hippocampal volumetry) is most practical. However, this first relies on establishing the reliability of automated tools.^136,140-142^ In the case of hippocampal volumetry, there has been successful translation into FDA-approved tools (e.g. NeuroQuant, which makes use of normative ranges to identify hippocampal atrophy in epilepsy patients), with similar (or slightly lower) specificity and sensitivity as neuroradiologists.^57,143,144^

In developing detection tools, the gold standard comparison is generally against visual assessment by expert neuroradiologists or epileptologists. When using imaging features that can be visually assessed, like hippocampal atrophy or hyperintensity, the added benefit of a novel diagnostic tool beyond what a clinician can already do is somewhat difficult to appreciate, particularly when performance is comparable. However, developing anomaly detection tools using quantitative MRI or using multi-modal approaches could provide substantial benefit. For example, T_2_-relaxometry for hippocampal sclerosis detection necessitates some form of comparison to interpret the quantitative signal in an individual—often by deriving asymmetry indices or by reference against a norm. As quantitative imaging tools increasingly deliver promise of improved detection of clinically relevant changes, the use of normative frameworks for such tools may be valuable.

The benefit of tools making use of normative models for detecting abnormalities is likely to be greatest for structural lesions that are difficult to visually detect; most notably, focal cortical dysplasias. Lesion detection has been a key focus for epilepsy neuroimaging researchers, inspiring the creation of the likes of the Multi-centre Epilepsy Lesion Detection (MELD) project,^145^ which has adopted numerous machine learning strategies (e.g. convolutional neural networks, support vector machines) to identify focal cortical dysplasias with varying performance.^67,68^ Thus far, the application of normative models in FCD detection have been limited. Outlier detection approaches based on normative modelling frameworks have been used for FCD detection from structural MRI features, demonstrating high sensitivity and specificity [area under the curve (AUC) of 0.96] in small samples.^146^ Similarly, FCD cases have been included within outlier detection tools using variational autoencoders to model normative features from diffusion MRI data.^147^ As these studies that have adopted normative modelling frameworks for lesion detection have only included small normative samples, it will be interesting to assess how these frameworks perform when larger and more diverse normative samples are included. More recent work making use of a generative manifold learning approach on T1-weighted images from a large normative dataset has indeed demonstrated promise for FCD detection.^148^

Of note, normative modelling approaches are inherently unsupervised approaches that do not rely on clinical labels, with the goal being to learn normal distribution for anomaly detection, rather than to learn the ‘abnormal’ feature. This is in contrast to many popularized methods for FCD detection, which make use of supervised learning approaches trained on radiologically defined lesions, meaning lesions should be able to be visually appreciated by a well trained neuroradiologist. It is important to highlight the difficulties of detecting such lesions visually (with >80% of FCD type I and over 30% of FCD type II demonstrating radiologically normal structural MRI^64^), and it is generally only along with the use of numerous imaging modalities, EEG, neuropsychological and clinical investigation that these lesions are often localized. Moreover, some pathological subtypes of FCD (e.g. FCD type I, FCD type IIA) are much more difficult to appreciate on structural MRI than are others with clear radiological features (e.g. grey-white matter blurring and transmantle sign in FCD type IIB). In this context, the use of diffusion MRI presents a promising avenue for further exploration, as it affords the potential of characterizing changes in tissue microstructure that likely aligns with pathological subtypes.^75,77^

Potential improvements for FCD detection will likely come from the following implementations: (i) making use of unsupervised learning approaches, including anomaly detection approaches; (ii) making use of quantitative imaging modalities that may better characterise subtle lesions; and (iii) integrating multi-modal approaches for lesion detection. To this end, normative modelling techniques may offer a promising solution (Fig. 4): these unsupervised approaches are well suited for characterizing distribution of quantitative data, while differentiating clinically meaningful changes from variability due to confounding effects like scanner or protocol differences.^149^ In addition, there is growing promise in the ability of normative models to integrate multi-modal data,^150^ which is likely to be particularly valuable for FCD detection.

#### Normative modelling for epilepsy prognosis and prediction

Going beyond focal abnormality detection for lesion localization, these normative frameworks could also be used to translate quantitative neuroimaging tools into prognostic or predictive biomarkers. Individuals’ outputs from normative modelling could become valuable clinical decision-making tools if they demonstrate predictive value, and their high interpretability makes them well suited for easy adoption into clinical practice.

Current decision-making and outcome prediction for epilepsy patients depends largely on subjective clinical judgement, potentially at the cost of improving overall outcomes.^151^ Neuroimaging data are often key to the clinical decision-making process, particularly in the context of surgical planning; however, much of the evidence supporting the predictive value of MRI on post-surgical outcomes stems from their inclusion as a binary feature in prediction models. For example, the presence or absence of hippocampal atrophy or sclerosis is often used to predict surgical outcome in patients with temporal lobe epilepsy, thanks to decades of evidence to support the high predictive value of abnormal MRI for favourable surgical outcome.^25,152-154^ Correspondingly, in the pre-surgical clinical work-up, clinicians may look for evidence or absence of MRI abnormality to guide their decision to recommend surgery or not. The use of normative modelling approaches could be valuable in this context: they may provide better indications of MRI abnormality than relying on qualitative assessments, and can be expanded into quantitative MRI tools.

Normative modelling frameworks could also be used to further improve outcome predictions if certain deviation patterns in epilepsy patients are shown to be associated with distinct outcomes. When benchmarking individuals against a normative reference, the individual level result is presented as a deviation score with respect to the norm. This could be a single summary deviation score for that individual (taking into account a range of features or modalities), defined per brain region within an atlas, or a map of deviation scores across the entire brain (Fig. 4). While such deviation scores or maps could be used to categorize patients as either exhibiting MRI abnormality or not (as a binary feature to predict outcomes), deviation patterns could also be directly used as inputs into prediction models for treatment outcome or efficacy. In doing so, we may be able to identify patterns of deviation that are predictive of a certain prognosis or outcome, or best suited to a particular treatment option. Of note, here, there is still an underlying assumption that specific patterns of deviation may be associated with a single outcome, which may not always be the case in heterogeneous disease. However, by using normative models that capture individual deviations while already accounting for other potential causes for heterogeneity (e.g. age, sex, genetic profile), we may be able to provide neuroimaging inputs that are more meaningfully linked to clinical outcomes into prediction models.

One key advantage of using these deviation scores in outcome prediction is that they are highly interpretable and explainable (they simply suggest a given individual is at a certain deviation or centile with respect to the norm). If indeed deviation patterns from normative models map meaningfully to specific outcomes, they could be very useful to clinicians as they could easily be integrated into existing decision-making processes in epileptology. Neuroimaging deviation patterns in themselves may be only partially predictive of clinical outcomes in epilepsy. However, normative modelling frameworks are well suited for benchmarking these brain changes relative to other, potentially more complex, variables (e.g. genetic, cognitive or behavioural information), meaning they could be rapidly expanded to integrate multimodal data, while still producing an interpretable output of individual deviation relative to the norm. In contrast, artificial intelligence (AI)-derived predictions are often not explainable in terms of the rationale for reaching a decision, and ‘black-box’ predictive algorithms are unlikely to be acceptable solutions to patients or clinicians in key healthcare decisions. Although there is enormous potential in the contribution of AI-based tools for epilepsy (as has been summarized in a number of excellent review papers^117,119,155,156^), interpretability and explainability are crucial for tools that directly impact decision-making. The importance of explainable AI (XAI) in healthcare is of growing interest, as AI-based clinical decision support systems will likely require explainability if they are to be widely adopted.^157^

### Key considerations and recommendations for future research

For normative modelling approaches to contribute to accelerated delivery of precision diagnosis and prediction in epilepsy, there are a number of key considerations for future research studies. First, it is important to identify the best (practical) imaging approaches that are likely to be sensitive diagnostic or prediction biomarkers for epilepsy. While the development of normative diffusion MRI approaches may be well suited for the specific task of improved FCD detection (given their sensitivity to tissue microstructure), the ability for diffusion MRI to be sensitive to pathology depends strongly on the way in which data are acquired and modelled. While the benefit may outweigh the practical costs of such approaches in the specific context of difficult-to-detect lesions, more practical solutions (e.g. using structural MRI-derived features) are likely to be valuable for the development of prognostic biomarkers.

Second, normative reference models should include diverse datasets to improve their potential generalizability. Here, it is important to consider geographically, ethnically and genetically diverse datasets, as well as diversity in data acquisition and scanner hardware. Carefully operationalizing inclusion and exclusion criteria for these normative cohorts is also likely to be important—if exclusion criteria are too exclusive, normal (healthy) variability may be underestimated, and the impact of prevalent comorbidities overlooked. Without diverse datasets, the generalizability of normative models for anomaly detection is likely to be limited. This also impacts the practicality of developing novel biomarkers—although advanced imaging modalities have demonstrated improved sensitivity for detecting subtle lesions, datasets including such techniques are less readily available, meaning that normative models for such data will be more difficult to develop. Moreover, if the required datasets are challenging to acquire, then clinical adoption is likely to be limited.

Of note, combining large, diverse datasets for the purposes of normative modelling, while increasing their potential generalizability, raises an additional challenge: that of data harmonization. Neuroimaging data are known to be highly dependent on differences in scanner hardware, acquired protocols and postprocessing pipelines, and although normative modelling approaches demonstrate promise for harmonizing site- or scanner-related effects (potentially to a greater degree than other statistical harmonization approaches),^149^ comparison of individual patients against normative references still largely relies on the inclusion of control data from the same site/scanner/protocol. In clinical settings, control datasets are often not available, or are limited to pseudo-controls, who can be painstakingly difficult to identify. The success of normative modelling solutions for clinical epileptology will therefore likely rely on improvements in data standardization and harmonization, as well as integration into scanner vendor systems at clinical centres.

In the context of novel precision biomarker development, validation will also need to be conducted with care. For diagnostic biomarkers of epilepsy-causing lesions, this is generally achieved through histopathological validation; however, histological findings are only available for patients who have undergone surgical resection (and control tissue rarely available), which can pose a challenge. Validation studies for novel diagnostic biomarkers is also particularly challenging in the context of heterogeneity, as it can be difficult to identify enough patients with shared imaging and pathological signatures for validation. Normative modelling approaches enable individual-level abnormalities to be identified, meaning that individualized, quantitative imaging features can be independently associated with pathological findings. The use of quantitative deviation scores may be a precise form of phenotyping MRI-related changes than current qualitative approaches, which could make them conducive to ‘deep phenotyping’ approaches for precision medicine.^158^

Finally, it is important for future studies to adopt standardized reporting strategies for model performance. Currently, researchers use variable measures to describe model performance (e.g. when using machine learning approaches for FCD detection), making it difficult to compare models to one another. Researchers should adopt recommended reporting strategies developed in the artificial imaging domain [such as the Checklist for Artificial Intelligence in Medical Imaging (CLAIM)^159^] to ensure better comparison across differing approaches. Researchers should report standardized performance measures, including model accuracy and AUC values. Moreover, validation should always be performed in an independent sample, whilst application to clinical cohorts should ideally include more than one site, with an emphasis on diversity and representativeness. Without this, we run the risk of the literature claiming to have identified precision biomarkers that are unable to be generalized for clinical use.^160^

## Conclusions

As we move toward identifying diagnostic and prognostic tools for personalized medicine in epilepsy, we require frameworks that are optimal for their development. Precision medicine relies on individualized approaches to treatment, considering the characteristics specific to the individual patient (e.g. age, sex, race, clinical features). Normative modelling approaches do just this: they account for parameters unique to an individual, and benchmark them against a normative reference set, enabling individual-level inference. By adopting these frameworks into research studies that test the diagnostic and prognostic accuracy of various neuroimaging techniques, we are likely to be able to identify important MRI biomarkers for precision epileptology. However, it will be key to carefully consider target imaging modalities best suited to the task, adopt standardized reporting strategies, while incorporating diverse datasets to ensure generalizability of these imaging tools for individual patient diagnosis.
